# Biological Function of Short-Chain Fatty Acids and Its Regulation on Intestinal Health of Poultry

**DOI:** 10.3389/fvets.2021.736739

**Published:** 2021-10-18

**Authors:** Lixuan Liu, Qingqing Li, Yajin Yang, Aiwei Guo

**Affiliations:** ^1^Faculty of Life Sciences, Southwest Forestry University, Kunming, China; ^2^Kunming Xianghao Technology Co., Ltd., Kunming, China

**Keywords:** short-chain fatty acids, biological function, regulation, intestinal health, poultry

## Abstract

Short-chain fatty acids (SCFAs) are metabolites generated by bacterial fermentation of dietary fiber (DF) in the hindgut. SCFAs are mainly composed of acetate, propionate and butyrate. Many studies have shown that SCFAs play a significant role in the regulation of intestinal health in poultry. SCFAs are primarily absorbed from the intestine and used by enterocytes as a key substrate for energy production. SCFAs can also inhibit the invasion and colonization of pathogens by lowering the intestinal pH. Additionally, butyrate inhibits the expression of nitric oxide synthase (*NOS*), which encodes inducible nitric oxide synthase (iNOS) in intestinal cells via the PPAR-γ pathway. This pathway causes significant reduction of iNOS and nitrate, and inhibits the proliferation of Enterobacteriaceae to maintain overall intestinal homeostasis. SCFAs can enhance the immune response by stimulating cytokine production (e.g. TNF-α, IL-2, IL-6, and IL-10) in the immune cells of the host. Similarly, it has been established that SCFAs promote the differentiation of T cells into T regulatory cells (Tregs) and expansion by binding to receptors, such as Toll-like receptors (TLR) and G protein-coupled receptors (GPRs), on immune cells. SCFAs have been shown to repair intestinal mucosa and alleviate intestinal inflammation by activating GPRs, inhibiting histone deacetylases (HDACs), and downregulating the expression of pro-inflammatory factor genes. Butyrate improves tight-junction-dependent intestinal barrier function by promoting tight junction (TJ) assembly. In recent years, the demand for banning antibiotics has increased in poultry production. Therefore, it is extremely important to maintain the intestinal health and sustainable production of poultry. Taking nutrition strategies is important to regulate SCFA production by supplementing dietary fiber and prebiotics, SCFA-producing bacteria (SPB), and additives in poultry diet. However, excessive SCFAs will lead to the enteritis in poultry production. There may be an optimal level and proportion of SCFAs in poultry intestine, which benefits to gut health of poultry. This review summarizes the biological functions of SCFAs and their role in gut health, as well as nutritional strategies to regulate SCFA production in the poultry gut.

## Introduction

Increasing evidence has revealed that the gut microbiota is a key contributor to health and gut homeostasis of the host. This positive effect may be achieved by producing short-chain fatty acids (SCFAs), which are the main metabolites produced by anaerobic bacteria in the hindgut by fermenting dietary fibers (DFs) (1). SCFAs can also be produced during protein fermentation, however, the main products of proteolysis have adverse effects on the host because they are related to carcinogenic and inflammatory activities. Importantly, SCFAs have been demonstrated to exert decisive effects on regulating the gut internal environment, improving the immune system, and inhibiting intestinal inflammation (2). The gastrointestinal tract (GIT), the largest organ of the host, is important for digestion and absorption of dietary nutrition (3), and also prevents the invasion and colonization of pathogens and toxins (4). Furthermore, a significant population of microbiota and immune cells are also present in the gut. Therefore, maintaining a healthy gut is important for overall health and enhanced productivity of poultry. In the context of banning antibiotics, nutrition regulation has become an effective strategy for maintaining intestinal health and reducing antibiotic use in poultry production (5). This paper reviewed the production pathways and biological functions of SCFAs, and poultry intestinal health, and the regulation of the production of SCFAs to maintain poultry intestinal health.

## Production of SCFAs

**Figure 1:**
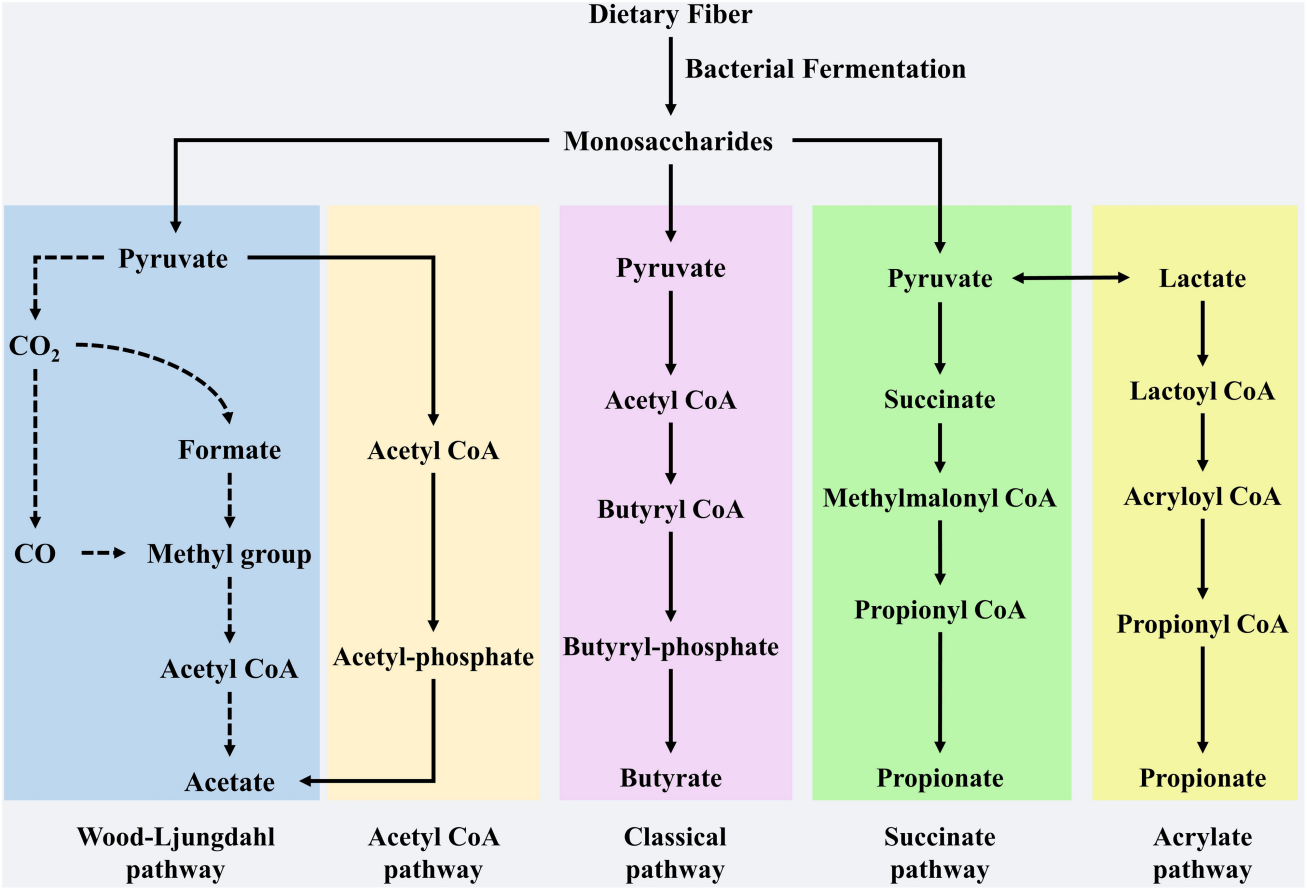
The synthesis pathway of SCFAs. Acetate, propionate and butyrate are main SCFAs produced by dietary fibers of microbial fermentation in gut. Production of acetate involves in pyruvate by Acetyl-CoA and Wood-Ljungdahl pathway. Butyrate is synthesized from Acetyl CoA that is subsequently reduced to Butyryl-CoA, which can be converted to butyrate by so-called classical pathway via Butyryl-phosphate. Propionate can be formed from succinate that is converted to methylmalonyl-CoA by succinate pathway. Furthermore, as a precursor lactate also participate the synthesis of propionate by acrylate pathway.

The gut microbiota is a complex community with hundreds of diverse microorganisms (6) which contributes to the breakdown of food and energy metabolism and affects the immune system and homeostasis (7). Gut microbes in the cecum ferment indigestible carbohydrates in food components to produce a series of metabolites (8). Among the various metabolites, SCFAs have received extensive attention because of their positive effects on health. SCFAs are defined as groups of fatty acids comprising less than six carbons, mainly acetate, propionate, and butyrate (9). These three fatty acids accounted for more than 95% of the total SCFAs, with a ratio of 60:20:20 (10). However, this proportion was not constant, as it relies on many factors, such as dietary components, microbiota composition, and the site of fermentation (11). Acetate is most abundant in the colon, accounting for more than half of the total SCFAs detected in feces (12) and can be formed through two major pathways: the acetyl-CoA and Wood-Ljungdahl pathways (13). *Bacteroides* spp., *Bifidobacterium* spp *Ruminococcus* spp., *Blautia hydrogenotrophica, and Clostridium* spp. are involved in these two pathways (14, 15). Aacetogenic bacteria can also synthesize acetate from carbon dioxide and formate through the Wood-Ljungdahl pathway (15). Propionate formation consists of two pathways: propionate can be produced by succinate, which involves the descarboxylation of methylmalonyl-CoA to propionyl-CoA (16). Firmicutes and Bacteroidetes participate in this pathway. Propionate can also be synthesized through the acrylate pathway, in which lactate is converted to propionate, however, only a few members of the family, such as Veillonellaceae and Lachnospiraceae, participate in this pathway (17, 18). Butyrate is produced from acetyl-CoA (the classical pathway) by several Firmicutes (19). Previous studies have indicated that many gut microbiota members, such as Actinobacteria, Proteobacteria, and Thermotogae, may be potential butyrate-producing bacteria, since these microbiotas contain vital enzymes, including butyryl coenzyme A dehydrogenase, butyryl-CoA transferase, and butyrate kinase, to synthesize butyrate (20). In addition, butyrate can also be synthesized from proteins through the lysine pathway, which demonstrates that gut microbiota can accommodate changes in the fermentation substrate, with the aim of retaining metabolite synthesis (20). The synthesis pathway of the SCFAs is shown in Figure 1.

Furthermore, protein fermentation in the hindgut can also produce SCFAs, as well as branched-SCFAs (BCFAs), such as 2-methylbutyric acid, isobutyric acid, and isovaleric acid. BCFAs are derived from branched-chain amino acids (such as valine, leucine and isoleucine) (21). Excessive amino acid or protein fermentation in the hindgut generates nitrogenous metabolites which can be harmful to gut integrity (22). Studies have demonstrated that the addition of dietary fiber [e.g. resistant starch 4 (RS4)] decreases the BCFAs (e.g. isobutyrate and isovalerate) in feces, and increases butyrate and total SCFAs. Prior research has shown that RS4 inhibits the fermentation of colonic proteins and reduces isobutyrate and isovalerate in feces, and increases butyrate and total SCFAs. The reduction of BCFAs in the colon indicates that it is beneficial for nutrient metabolism and gut health (23, 24). Nonetheless, there is a contradictory view of BCFAs in that *IL-18* mRNA expression in intestinal epithelial cells can be inhibited by BCFAs, which can also ease the inflammatory response to lipopolysaccharide (LPS) challenge (25).

## Biological Function of SCFAs

Current research shows that SCFAs have important biological functions in the body. SCFAs play a major role in the host energy metabolism. Although acetate and propionate can perform a certain degree of energy metabolism, most studies have focused mainly on the role of butyrate. About 70–80% of butyrate are metabolized by colon cells, thus promoting colon cell growth and function (26). In the colon cells of GF mice, energy deprivation (a state of decreased enzymes in the tricarboxylic acid cycle) resulted in reduced ATP levels, while using butyrate-producing bacteria to colonize GF mice and butyrate to treat GF mice colonocytes *ex vivo* contributes to an increase in oxidative phosphorylation, suggesting the importance of butyrate and butyrate-producing bacteria in colonocyte energy metabolism (27). Butyrate also improves body gain and fat mass induced by a high-fat diet (HFD). Supplementation with 5% sodium butyrate (SB) in food can effectively decrease body gain in obese mice (28). Another research group reported that after feeding mice with a butyrate-enriched diet, the level of energy expenditure considerably increased, which reduced the prevalence of obesity (29). The positive effect of butyrate on metabolic changes induced by HFD appears to depend on the down-regulation of *PPAR*γ (per-oxisome proliferator-activated receptor), which promotes the switch from lipid synthesis to lipid oxidation (28). SCFAs can regulate hormones involved in appetite regulation. After infusing SCFAs into the rat colon, the gut transit rate and appetite are significantly decreased; however, the concentration of PYY in the peripheral circulation is increased. These findings suggest that the inhibitory effect of SCFAs on gut motility and appetite may be mediated by PYY (30). Glucagon-like peptide-1 (GLP-1) may cause a decrease in appetite and food intake (31) because higher levels of GLP-1 in the systemic circulation are positively correlated with slower gastric emptying time (32). Acetate can induce the expression of neuropeptides such as proopiomelanocortin (POMC) and agouti-related peptide (AgRP) to regulate appetite and reduce food intake via a central homeostatic mechanism (33). Moreover, SCFAs modulate the expression of leptin, which is secreted by adipose tissue, to decrease food intake. Butyrate has been shown to promote leptin production in adipose tissue by activating GPR41 and GPR43, thus reducing food intake and controlling weight gain (34).

## SCFAs and Poultry Gut Health

### SCFAs and Gut Barrier

The gut barrier separates the intestine from the body and exerts immunity and physical defense against pathogens, viruses, and environmental toxins. Four interlinked and interdependent barriers collectively form an intact gut barrier. These include the microbial barrier, chemical barrier, mechanical barrier, and immunologic barrier. The microbial barrier refers to various microorganisms attached to the intestinal surface. Here, there is competition between beneficial microbiota and pathogens for nutrients and attachment sites (35). A study indicated that acetate may play a critical role in the competitive process between *bifidobacteria* and enteropathogens, which helps to build a balanced gut microbiological environment (36). It was reported that the colonization of *Salmonella enterica* and *Clostridium perfringens*, driver of necrotic enteritis, were inhibited by butyrate in chickens gut (37, 38). Further an increase in some beneficial microbiota (such as *Christensenellaceae, Blautia* and *Lactobacillus*) was also reported after SB intervention (39). It is worth noting that changes in chicken gut microbiota induced by SCFAs were related to the reduced inflammatory response as SCFAs can reduce levels of LPS - a main component of Gram-negative bacteria and stimulater of inflammation response. SCFAs also indirectly regulate the gut microbiota microbial barrier by promoting secretion of mucins and antimicrobial peptides in the gut (40).

The gut chemical barrier, the so-called mucus layer, consists mainly of a layer of mucus covering the intestinal epithelial cells, which plays an important and unique role in maintaining intestinal barrier function and homeostasis. Mucins (high molecular weight glycoproteins) that are synthesized and secreted by goblet cells are the main components of the chemical barrier (41, 42). The produced mucins are stored in the goblet cell cytoplasm in the form of granules and are transported to the cell surface (43). The importance of mucus on gut health is reflected in the following aspects: (1) It forms a skin covering the intestinal cells, which protects against microbes, for example, colonization and invasion of pathogens is facilitated in mice lacking a mucus layer compared with wild type mice (44, 45); (2) it is characterized by moisturizing and lubricating properties that protect intestinal cells from dehydration when they pass through the lumen (46); (3) it plays a role in immune functions. A study indicated that mucins bind to luminal antigens, particularly bacteria, and are associated with galectin-3 to inhibit inflammatory responses (47). Under normal conditions, mucins are constantly produced by goblet cells, whereas SCFAs can regulate this process by affecting mucin gene expression. A study showed that when butyrate was used as the sole carbon source, the production of Muc2 was increased significantly in the colon, which may rely on GPR109A in goblet cells (48). Adding a mixture of butyrate and acetate into drinking water can significantly improve the gut chemical barrier function of mice with colitis by enhancing Muc2 gene expression (49). Hansen et al. (50) indicated that supplementation tributyrin as a therapeutic measure can significantly increase ileum Muc2 mRNA expression in coccidia-infected broilers. Interestingly, the regulation of mucin production by SCFAs does not appear to be dose-dependent; lower concentrations of SCFAs will dramatically increase Muc2 levels in mice, however, higher concentrations had the opposite effect (51). Similarly, an *in vitro* study showed that butyrate (0.05–1 mmol/L) could stimulate the synthesis of Muc2 in normal colon tissue, while 10 mmol/L butyrate could restore the synthesis of Muc2 to the basic level (52), likely because of the induction of apoptosis at higher butyrate levels. Although the mechanism by which SCFAs regulate Muc2 production remains unclear, some studies have shown that SCFAs can induce *Muc2* transcription by AP-1 binding and histone acetylation at the *Muc2* promoter (51). These results indicate that SCFAs regulate the gut chemical barrier mainly by promoting the levels of Muc2, thereby enhancing the host's resistance to foreign pathogens.

The gut's mechanical barrier is a layer of polarized cells consisting of intestinal epithelial cells and stem cells, goblet cells, and Paneth cells (53). These cells are linked by tight junctions (TJ), adhesion junctions (AJ), gap junctions, and desmosomes, which decrease gut permeability and maintain gut mechanical barrier stability (54). Propionate promotes the production of related proteins consisting of tight junction proteins ZO-1 and occludin, thus improving gut barrier function (55). When pigs received gastric infusion of SCFA, the gene expression of *occludin* and *claudin* was increased, which positively improved the gut mechanical barrier (42). Butyrate as a preventive treatment can effectively decrease gut permeability characterized by reduced concentrations of D (–)-lactate in DSS-induced broilers, and this beneficial changes are closely related to restoration of tight junction function (56). Although underlying mechanism of butyrate to enhance tight junction function is not clear, a study indicated that MLCK (Myosin Light Chain Kinase) may play an important role in regulating tight junction (57). Research showed that tributyrin can enhance cell tight junction and reduce intestinal permeability by promoting mRNA relative expression of mucosal barrier related genes, such as occludin, claudin-1, claudin-4 and JAM-3 (junctional adhesion molecule-3), in coccidia-infected broilers (50, 58). In addition, dietary supplementation with *Clostridium butyricum* improves growth performance and intestinal mechanical barrier by upregulating various genes including claudins 2, 15, 19, and 23, tight junction proteins 1, 2, and 3 in broilers (59). The studies demonstrated that butyrate-producing bacteria or butyrate can also play a same role in supplying butyrate to improve gut barrier of poultry. In brief, regulation of SCFAs on the gut mechanical barrier is mainly through promoting the gene expression of relevant junction tight proteins, thereby reducing intestinal permeability and improving the animal's gut barrier functions.

The gut immunologic barrier consists of various immune cells localized in the epithelium or below the intestinal epithelial cells (IECs), such as T cells, B cells, macrophages, and dendritic cells, which collectively protect against pathogen invasion and maintain gut health (60). Macrophages can be recruited to the injured intestinal wall to promote the division and growth of intestinal epithelial cells (61). Regulation of SCFAs on gut immunity mainly relies on the differentiation and recruitment of immune cells and downstream expression of immune factors. SCFAs play an important role in inhibiting gut inflammation, as they can induce T cells to differentiate into Tregs (62). The differentiation and metabolism of macrophages and an enhancement of antibacterial peptide gene expression are attributed to butyrate regulation (63). Butyrate independently regulates IgA production and assists in the increased expression of IL-10 (62).

### SCFAS and Gut Microbiota

The microbial barrier, composed of intestinal microorganisms, is an important part of the intestinal barrier. One crucial property of gut microbiota in poultry is to establish colonization resistance against pathogen invasion by fermenting non-digestible carbohydrates into SCFAs. SCFAs can release H^+^ and decrease the pH of the hindgut, which inhibits pathogen invasion and colonization. SCFAs (mainly butyrate) consume luminal oxygen to create an anaerobic environment, thereby reducing aerobic pathogens such as *Salmonella* expansion in the gut lumen (64). Studies have demonstrated that SCFAs can inhibit colonization by several pathogens, such as *Salmonella* spp., *Escherichia coli*, and *Shigella* spp., to maintain a stable microbial environment. SCFAs play an inhibitory role in the colonization of pathogens such as *Escherichia coli* and *Shigella*, thus contributing to gut protection (36). Propionate inhibits *Salmonella typhimurium* proliferation by disrupting intracellular pH homeostasis (65). Experiments have shown that supplementation with 0.25–0.7% butyrate, formate, and caproate significantly decreased the abundance of *Salmonella typhimurium* and *Salmonella enteritidis* in the cecum of broilers and piglets (37, 66–68). In addition, studies have indicated that the abundance of *Escherichia coli* in broiler chicken crops of butyrate-treated groups (0.2, 0.4, and 0.6%) decreased significantly, whereas that of *Escherichia coli* in the small intestine and cecum decreased in butyrate-treated groups (0.4 and 0.6%) compared to 0.2% butyrate and control groups (69). SCFAs promote the proliferation of some beneficial bacteria in poultry, such as *Bifidobacteria* and *Lactobacilli*. These beneficial bacteria in turn stimulate SCFA synthesis, while produced SCFAs not only reduce luminal pH and inhibit pathogen colonization, but also improve gut microbial barrier function contributing to gut homeostasis. Dysbiosis of gut microbiota is controlled by several factors, including antimicrobial peptides and immunoglobulins, and leads to a series of gut diseases such as intestinal inflammation (70). Interestingly, butyrate may induce a positive alteration in gut microbiota composition (71). Butyrate can regulate the expression of antimicrobial peptides that are involved in the control of gut microbiota composition (72) and can induce the production of IL-18, which regulates gut microbiota composition and antimicrobial peptides (73). Butyrate can promote the production of secretory immunoglobulin A (sIgA) cells by activating B cells, while sIgA is linked to the composition of the gut microbiota (74). In summary, the interaction between butyrate and many factors positively contributed to the gut microbiota composition, thereby maintaining microbial homeostasis.

### SCFAs and Gut Immune

It is difficult to understand the effects of SCFAs on intestinal immunity because of the complex interaction with multiple signaling molecules. There are two main mechanisms involved in the regulation of host health or disease by SCFAs. One is the regulation of target cell epigenetics after SCFAs enter the cells, such as the inhibition of HDACs via SCFAs regulating the expression of relevant genes (75). SCFAs as a signal molecule combine with GPRs and then play an important role in the regulation of various host physiology (76). Furthermore, the activator and expression of GPRs is dissimilar. The most powerful activator for GPR43 mainly expressed in immune cells, enteroendocrine cells and adipocytes is propionate (77) and the order of ability to activate GPR41 that is widely expressed in adipose, spleen as well as colon is: propionate > butyrate > acetate (78, 79), yet GPR109A highly expressed in adipocytes, immune cells, and colon is only activated by butyrate at low level (80). Many studies have verified the effect of SCFAs on immunoregulation by the activation of GPRs and regulation of cytokines. A study conducted by (81) revealed that IL-22 production was elevated by the activation of GPR41 via SCFAs, which promotes gut homeostasis and protect against inflammation. Another researcher (82) demonstrated the importance of SCFAs to active GPR43 on the production of microbiota antigen-specific Th1 cell IL-10 production, in addition they also indicated that Gpr43^**−/−**^ mice showed a severe intestinal inflammation induced by DSS (Dextran Sulfate Sodium) than wild-type mice. Butyrate combines with GPR43 to stimulate potassium ion flow, which leads to a hyperpolarisation of the intestinal epithelial cell membrane, activation of the NLRP3 inflammasome, and upregulation IL-18, thus maintaining intestinal epithelial integrity and mucosal homeostasis in mice with colitis (73). Moreover a research has also proven the ability of butyrate to alleviate gut inflammation by differentially controling differentiation of Th1 and Th17 and enhancing IL-10 production (83). In addition to GPRs, SCFAs also regulate the immune response by inhibiting HDAC activity. A study has reported the effect of SCFAs on immunoregulation by inhibiting HDAC (84). The effective effect of butyrate to protect against gut inflammation by inhibiting HDAC in macrophages and dendritic cells indicated that butyrate can down-regulate pro-inflammatory cytokines e.g. IL-1β, IL-6, and IL-8 by the inhibition of HDACs, which regulates macrophage function (85, 86). In addition, a series of recovery effect of gut inflammation, such as decreased TNF-α level, suppressed *NF-*κ*B* activity, and elevated IL-10 concentrations, were ovserved in mononuclear cells and neutrophils by inhibition of HDACs via butyrate and propionate intervention (87). Foxp3 (forkhead box P3) is a characteristic marker molecule that maintains Treg function, and HDACs can cause degradation of Foxp3 by affecting its deacetylation level, which affects the immunity homeostasis caused by the dysfunction of Treg function (88). This dysfunction can decrease the expression of HDACs in Tregs in mice and increase the degree of histone acetylation, thus promoting Treg differentiation and overall enhancement of the immune response (89). To conclude, SCFAs can regulate the production of inflammatory cytokines to improve immunity by inhibiting HDACs and activating GPRs.

### SCFAs and Gut Inflammation

The inflammatory response is a natural defensive mechanism that protects against pathogen infection. Under normal circumstances, immune cells recruit and secrete pro- or anti-inflammatory cytokines to protect the body from damage when pathogens invade a particular site. However, when the balance between immune and inflammation is altered, a serious inflammatory response can arise and lead to the development of pathological diseases by the secretion of pro-inflammatory cytokines (90). As an important immune organ in the host, the gut is easily affected by inappropriate inflammatory activation caused by feed, environment, bacteria, and metabolites (91). Therefore, effective approaches to regulate pathogenic factors may be used to relieve intestinal inflammation. The levels of pro-inflammatory cytokines, such as IL-6, IL-1β and TNF-α, were decreased in pathogen-free chickens with *Salmonella* infection after inulin supplementation, which may be related to SCFA production (92). In a *Clostridium difficile*-induced colitis mice model, butyrate alleviated gut inflammation and reduced gut permeability by steadying HIF-1 (hypoxia inducible factor) and increasing tight junctions (93). Another study has shown that propionate has protective effects in mice with colitis by the regulation of expression of Reg3 mucosal lectins (94). The positive effect of SCFAs on intestinal inflammation has been proven in SCFA-receptor knockout inflammation model mice (95). Moreover, compared to wild-type mice, GPR43^−/−^ mice show a stronger reaction to the LPS infection characterized by greater intravascular neutrophil rolling and adhesion (96). The production of several cytokines induced by SCFAs may be involved in the inflammatory response. In particular, GPR43 and GPR109A activation by acetate and butyrate, respectively, may suppress the inflammatory response, which is completed by avoiding chemotaxis of monocytes to the inflammatory site (97) and enhancing the production of chemokines and cytokines (98). GPR109A also contributes to preventing the deterioration of colitis (99). A mouse model has shown that GPR43 and GPR109A activated by SCFAs can induce the NLRP3 inflammasome to promote the production of IL-18 (73). IL-18 promotes the expression of anti-microbial peptide and alleviates colitis (100). Oral administration of butyrate alleviates colitis in mice by promoting Treg cell differentiation (101), while butyrate enemas inhibited *NF-*κ*B* activation in mice with colitis (102). These results indicate that SCFAs can bind with GPRs to regulate the inflammatory response, providing sufficient evidence for the inhibition of intestinal inflammation by the SCFAs-depended GPRs pathway. Even though a previous study showed that GPR43-deficient mice are at a lower risk of developing chronic colitis than normal mice (103). HDACS counterweight the acetylation level of histones and influence the expression of many genes in intestinal epithelial cells and immune cells (104). The inhibition of HDACs is mainly attributed to an increase in acetylation of specific lysine residues in histones, thus promoting gene transcription, while inhibited HDACS are related to decreased production of pro-inflammatory cytokines (70). Some studies have indicated that the development of colitis is related to higher expression of HDACS9 in the inflammatory area, however HDACS9-deficient mice are not influenced by induced colitis (105, 106). In addition, HDACS2-deficient mice show stronger resistance to DSS-induced colitis (107). SCFAs (mainly butyrate), a histone deacetylase inhibitor (HDACSi), have been shown to significantly inhibit the production of pro-inflammatory cytokines e.g. IL-6 and IL-12 in intestinal epithelial cells as well as immune cells, thus alleviating gut inflammation (108). *NF-*κ*B* regulates the release and synthesis of inflammatory cytokines (109). HDACSi can reduce the inflammatory reaction by inhibiting *NF-*κ*B* activation and blocking nuclear translocation (70). SCFAs, especially butyrate, can inhibit the *NF-*κ*B* pathway, which effectively downregulates the expression of a series of pro-inflammatory cytokines in broilers, including IL-6 and TNF-α (110). Adding butyrate to the piglet diet can reduce the concentration of TNF-α and IL-6 in serum, thereby weakening the function of *NF-*κ*B* in the gut and inhibiting colonization of pathogens (111). Thus, the inhibitory effect of HDACSi on *NF-*κ*B* is involved in its anti-inflammatory effect (112). Regulation of mammalian target of rapamycin (mTOR) by SCFAs is related to the inflammatory response. SCFAs can regulate the mTOR pathway to increase T cell secretion of IL-10 and promote T-cell differentiation into Treg and Th cells by inhibiting HDACs, which plays an effective anti-inflammatory role (62). In summary, as an inhibitor of HDACs, SCFAs can alleviate a variety of intestinal inflammatory responses by regulating multiple pathways. This field may be a hot area for poultry nutrition in the future. However, there is a controversial result of SCFAs on the gut inflammation regulation, although SCFAs (especially butyrate) have been examined to prevent inflammation (113). The studies showed that the activation of GPR41/43 by SCFAs resulted in the activation of downstream mTOR, PI3K, or MAPK signaling pathways, thus exerting pro-inflammatory effects (114, 115). Acetate seems to involve in the production of pro-inflammatory cytokines and chemokines (e.g. IL-6, CXCL1, and CXCL2) by the activation of GPR41 or GPR43 and downstream signal pathway of extracellular signal-regulated kinase 1/2 (ERK1/2) and MAPK/p38 (116). Potential mechanisms for the opposite outcome of SCFAs in inflammation require further investigation. According to current reports, we speculate that it may be associated with variation in gut microbiota composition and the SCFAs concentrations (117).

**Figure 2:**
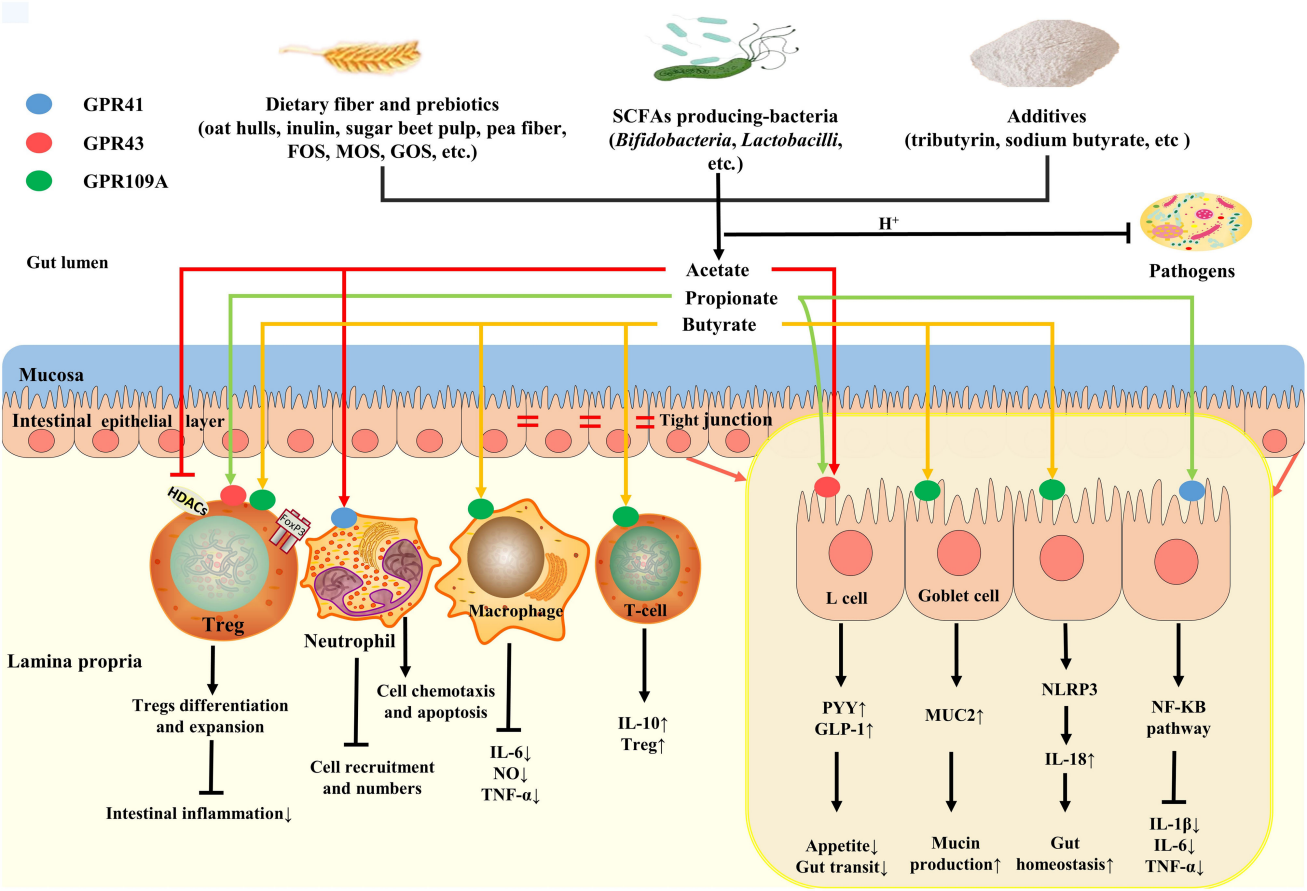
Roles and potential benefits of SCFAs on gut health of poultry. Dietary fibers, SCFAs-producing bacteria and additives collectively regulate SCFAs production in gut. SCFAs (mainly acetate) can reduce luminal pH, thus inhibiting pathogens colonization. Butyrate, as the major energy source, not only provide energy for epithelial cells metabolism, but also promote mucin production by binding to GPR109A in goblet cell, thus improving gut mucosa barrier. In addition, butyrate can play an anti-inflammatory and immunoregulatory effect by GPRs or HDAC inhibition, which maintains gut homeostasis. Luminal acetate or propionate sensed by GPR41 and GPR43 sited on L cells releases PYY and GLP-1, affecting appetite and gut transit.

In conclusion, the SCFAs have great significance to the intestinal health of poultry. The role and potential benefits of SCFAs on poultry intestinal health and the regulation of SCFAs production are shown in Figure 2.

## Regulation of SCFAs Production in Intestinal Tract of Poultry

### Dietary Fiber and Prebiotics

Dietary fiber is a comprehensive topic, so the effect of gut microbiota on dietary fiber varies depending on the type of fiber. In general, gut microbiota can ferment soluble dietary fiber to produce metabolites, such as SCFAs. Soluble dietary fiber mainly includes β-glucan, pectin, and arabinoxylans, which are usually found in oats, barley, apples and carrots (118). Dietary fibers that promote SCFA production have been widely reported. The effects of cassava root chips on SCFAs in broiler chick cecum showed that acetate, butyrate, and total SCFA contents were highest at 25% inclusion, and propionate content was highest at 37.5% inclusion (119). Research conducted by (120) showed that *Sarcodiotheca gaudichaudii* and *Chondrus crispus* significantly elevated acetate production (52.21 and 51.53 mmol/kg, respectively) (*P < 0.05*) in layer hens cecum, compared with the control group (29.94 mmol/kg). Walugembe et al. (121) indicated that two mixtures of wheat bran and DDGS (60 and 80 g/kg, respectively) resulted in an increase in total SCFAs and acetate concentrations in broiler and layer cecum. Prebiotics can also help to produce SCFAs in the gut of poultry. Song et al. (92) demonstrated that supplementation with inulin (1%) markedly increased acetate and butyrate concentrations in the cecum of chickens infected with *Salmonella* (*P* < *0.05*), while 1% inulin significantly decreased propionate concentrations (*P* < *0.05*). A similar study also indicated that different doses of inulin (0.25, 0.5, 1.5, and 2%) increased acetate concentrations in the chicken cecum, however, butyrate concentrations in the cecum were higher at doses of 0.5, 1.5, and 2% (122). A study showed that supplementation with 0.3% isomalto-oligosaccharides significantly increased butyrate and isobutyrate in the jejunum of broilers (*P* < *0.05*) (123). Research has also indicated that 0.05% xylooligosaccharides simultaneously stimulate acetate and butyrate production in the cecum of laying hens, but the propionate content was not affected by xylooligosaccharides (124). These findings suggest that the structure and chemical properties of dietary fibers are associated with the metabolism of the gut microbiota, and that specific feeding pattern and doses may impact SCFA production.

### SCFAs-Producing Bacteria

SCFA-producing bacteria (SPB) are known to be involved in the fermentation of dietary fibers by partially converting sugars, proteins, and peptides to SCFAs (22, 125). Considering that the production of SCFAs is closely related to intestinal microbes, it may be feasible to control the production of SCFAs by adding SPB.

Numerous studies have also demonstrated the positive effect of probiotics in promoting the production of SCFAs. Supplement *Lactobacillus salivarius* ssp. (10^7^ CFU/g) can significantly increase the concentrations of propionate and butyrate in broiler cecum (126). A study revealed that supplementation with *Clostridium butyricum* (2.5 × 10^9^ CFU/kg) in broiler chickens had a tendency (*P* = *0.063*) to reduce cecal propionate contents (8.90 to 8.07 μmol/g) compared with the control group (127). Effects of *Bacillus subtilis* PB6 (0.5 g/kg) on cecal SCFAs contents in broiler chicks is effective, because it significantly increased acetate and butyrate contents (*P* < *0.05*) (128). The effects of supplementation (10^7^ CFU/g) of multi-strain probiotics (*L. acidophilus* LAP5, *L. fermentum* P2, *P. acidophilus* LS, and *L. casei* L21) on SCFA production in young chicken cecum have also been reported (129). Their results showed that the contents of total SCFAs and acetate were elevated in the multi-strain probiotic-treated group than in the control group, however, the contents of butyrate and propionate were not different from those in the control group. Kan et al. (130) investigated the effects of *Bacillus licheniformis* on SCFAs content, and found that 3.2 × 10^9^ CFU/kg *Bacillus licheniformis* resulted in a significant increase in formate in the cecum of broilers (*P* = *0.027*). In conclusion, the effects of probiotics on SCFAs production have been proven. However, there were fewer researches of probiotics on SCFAs production in poultry and fewer studies about combination of probiotics. In future studies, more attention should be paid to the effect of the combined use of probiotics on the formation of SCFAs in poultry, so as to exert the great potential of probiotics.

### Additives

Current studies have shown that directly supplying short acids and short acid salts to poultry diets can increase the amount of SCFAs in the hindgut. At present, the common additives in poultry diets are butyrate, SB, tributyrin, propionate, and caproate. However, directly adding butyrate into feed faces huge challenges as butyrate has an unpleasant taste, potentially labile volatility, and high cost (131). To solve these problems, an increasing number of studies have focused on the regulation of SCFAs additives, such as SB and organic acids (OA), on butyrate production in the gut. Wu et al. (132) indicated that high level of SB (1 g/kg) significantly increased (*P* < *0.05*) concentrations of propionate and butyrate in broiler jejunum, while medium level (0.8 g/kg) and low level (0.4 g/kg) contributed to an increase in butyrate and acetate, respectively. González-Ortiz et al. (133) showed that 1 g/kg SB not only significantly increased lactate and acetate concentrations (*P* < *0.05*) in broiler chicken ileum, but also elevated butyrate contents in the jejunum. Wang et al. (134) showed that SB (20 and 40 g/kg) increased the contents of acetate, butyrate, and isobutyrate in laying hen cecum. Furthermore, some studies have also reported the effects of OA on SCFA production in the gut. A study showed that the use of 2 g/kg OA composed of acetic acid, formic acid, propionic acid, sorbic acid, and vegetal fatty acids can increase propionate and butyrate concentrations in the cecum of turkey (135). Alkhulaifi et al. (136) demonstrated that supplementation with 3 g/kg mixture (SCFAs, medium-chain fatty acids, and β*-*1,4 mannobiose) significantly increased the concentration of acetate and butyrate in the cecum of broilers (*P* < *0.05*). Butyrin also serves similar functions to affect SCFA production in the gut, but most research has focused on rodents and mammals. A previous study showed that acetate content in the cecum of weaned pigs challenged with LPS was elevated after tributyrin supplementation (2 g/kg) (137). Although tributyrin (4 g/kg) had no significant effects on SCFA concentrations in the piglet cecum, it tended to increase the concentrations of acetate (*P* = 0.53), propionate (*P* = 0.86), and butyrate (*P* = 0.59) (138). However, a study showed an inverse result, which showed that 5 g/kg monobutyrin decreased acetate, propionate, and isovalerate concentrations in rat cecum, while 5 g/kg tributyrin decreased valerate and isobutyrate contents (139). At present, nutritionists are paying increasing attention to improving the intestinal health of poultry via nutritional strategies. It is important to regulate the production of SCFAs in the hindgut by supplementing dietary fiber and prebiotics, SCFA-producing bacteria, and additives to poultry formulations. This will help maintain the intestinal health of poultry and alleviate intestinal inflammation, so as to maintain the healthy and sustainable development of the poultry industry after banning antimicrobial growth promoters (Table 1).

**Table 1:** Effects of dietary fiber, prebiotics, probiotics and additives on SCFAs production.

**Ingredient**	**Inclusion level**	**Specie**	**Age, d**	**Sample site**	**Effects**	**Reference**
Wheat bran + DDGS	80 g/kg	Broiler	0-21d	Cecum	↑Total SCFAs ↑Acetate	Walugembe et al., (121)
	60 g/kg	Laying hen				
Inulin	20 g/kg	Broiler	0-40 d	Cecum	↑Propionate ↑Butyrate	Li et al., (140)
Wheat bran	100 g/kg				↑Isobutyrate	
Inulin	10 g/kg	Chickens	0-56 d	Cecum	↑Acetate ↑Butyrate ↑Propionate	Song et al., (141)
Isomalto-Oligosaccharide	3 g/kg	Broiler	0-56 d	Jejunum	↑Isobutyrate ↑Butyrate	Zhang et al., (123)
Xylooligosaccharides	0.5 g/kg	Laying hen	0-56 d	Cecum	↑Acetate ↑Butyrate	Ding et al., (124)
Soybean oligosaccharide	6 g/kg	Broiler	0-49 d	Cecum	↑Acetate ↑Propionate	Zhu et al., (142)
Stachyose						
Raffinose						
*Bacillus subtilis* PB6	0.5 g/kg	Broiler chick	0-40 d	Cecum	↑Acetate ↑Butyrate	Aljumaah et al., (128)
*Clostridium butyricum*	1 × 10^9^ CFU/kg	Broiler chicken	0-42 d	Cecum	↑Acetate ↑Propionate ↑Total SCFAs	Zhang et al., (143)
*Clostridium butyricum*	2.5 × 10^9^ CFU/kg	Broiler chick	0-37 d	Cecum	↓Propionate	Molnár et al., (127)
*Lactobacillus plantarum* B1	2 × 10^9^ CFU/kg	Broiler chicken	0-42 d	Cecum	↑Acetate ↑Butyrate ↑Lactate	Peng et al., (144)
Multi-strain probiotics(*L. acidophilus* LAP5, *L. fermentum* P2, *P. acidophilus* LS, *L. casei* L21)	1 × 10^7^ CFU/g	Chickens	0-10 d	Cecum	↑Acetate ↑Total SCFAs	Chang et al., (129)
Sodium butyrate	1 g/kg	Broiler chicken	0-42 d	Jejunum	↑Butyrate	González-Ortiz et al., (133)
				Ileum	↑Acetate ↑Lactate	
Sodium butyrate	1 g/kg	Broiler	0-42 d	Jejunum	↑Propionate ↑Butyrate	Wu et al., (132)
Sodium butyrate	20 g/kg	Laying hen	0-28 d	Cecum	↑Acetate ↑Butyrate	Wang et al., (134)
	40 g/kg					
Organic acids(acetate, formate, propionate, sorbate, vegetal fatty acids)	2 g/kg	Turkey	0-70 d	Cecum	↑Propionate ↑Butyrate	Milbradt et al., (135)
Organic acid blend(SCFAs, MCFAs, β1-4 mannobiose)	3 g/kg	Broiler	0-35 d	Cecum	↑Acetate ↑Butyrate	Aljumaah et al., (136)

## Conclusions and Future Perspectives

In conclusion, SCFAs are important biomarkers to monitor intestinal health of poultry. SCFAs can improve barrier function, regulate microorganisms, enhance immune function and prevent inflammation of gut in poultry. The influence of dietary factors, through dietary interventions such as increasing dietary fiber, butyric acid, and acid-producing bacteria, can increase the SCFAs in the intestine, however, excessive SCFAs (butyrate and acetate) in the hindgut will promote the development of metabolic syndrome via the gut microbiota–brain–β-cell axis. So, an appropriate amount of SCFAs in the intestine may be beneficial to poultry gut health. The content and ratio of SCFAs in the intestine are affected by the characteristics of dietary fibers (solubility and fermentability of dietary fibers), undigested protein, and lipids. Therefore, there may be a threshold for SCFAs in the intestine. In the future, it is necessary to study how to regulate the production of SCFAs through nutritional strategies and determine the optimal level and proportion of SCFAs for maintaining intestinal health and preventing enteritis in poultry production after the banning of antibiotic.

## Author Contributions

LL wrote this review manuscript. YY collected literature. QL and AG reviewed the manuscript and given critical suggestions and comments. All authors have read and approved the final manuscript.

## Funding

This work was supported by the National Natural Science Foundation of China [Grant Numbers: 31860650 and 31460609]; the Scientific and Technological Innovation Team Construction Project for Protection and Utilization of Under-forest Biological Resources in Universities of Yunnan Province.

## Conflict of Interest

QL was employed by company Kunming Xianghao Technology Co., Ltd. The remaining authors declare that the research was conducted in the absence of any commercial or financial relationships that could be construed as a potential conflict of interest.

## Publisher's Note

All claims expressed in this article are solely those of the authors and do not necessarily represent those of their affiliated organizations, or those of the publisher, the editors and the reviewers. Any product that may be evaluated in this article, or claim that may be made by its manufacturer, is not guaranteed or endorsed by the publisher.
